# Antimicrobial prescribing practices and factors associated with antimicrobial prescribing in hospitalized COVID-19 patients in Oyo state: A retrospective study

**DOI:** 10.1371/journal.pgph.0003911

**Published:** 2024-11-13

**Authors:** Amos Abimbola Oladunni, Sina-Odunsi Ayomide Busayo, Yusuff Adebayo Adebisi, Rebecca Folasade Bamidele, Abila Derrick Bary, Oluwatoyin Elizabeth Afolabi, Attaullah Ahmadi, Michael Obaro, Don Eliseo Lucero-Prisno III

**Affiliations:** 1 Department of Pharmacology and Therapeutics, College of Medicine, University of Ibadan, Ibadan, Oyo State, Nigeria; 2 Department of Pharmacy, Afe Babalola Multisystem Hospital, Ado-Ekiti, Ekiti State, Nigeria; 3 Department of Pharmacology and Toxicology, College of Pharmacy, Afe Babalola University, Ado-Ekiti, Ekiti State, Nigeria; 4 Centre of African Studies, University of Edinburgh, Edinburgh, United Kingdom; 5 Nuffield Department of Population Health, University of Oxford, Oxford, United Kingdom; 6 Department of Medical Microbiology, University College Hospital, Ibadan, Oyo State, Nigeria; 7 College of Health Sciences, Makerere University, Kampala, Uganda; 8 College of Pharmacy, Afe Babalola University, Ado-Ekiti, Ekiti State, Nigeria; 9 Ecole des Hautes Etudes en Sante Publique, Paris, France; 10 Department of Global Health and Development, London School of Hygiene and Tropical Medicine, London, United Kingdom; Tunisian Institute of Veterinary Research, TUNISIA

## Abstract

**Background:**

Proportion of hospitalized COVID-19 patients receiving antimicrobial drug is significantly high despite evidence of low level of actual bacterial co-infection, potentially contributing to poor health outcome and global antimicrobial resistance.

**Materials and methods:**

A retrospective study was performed on antimicrobial agents prescribed to adult patients with confirmed COVID-19 admitted across three isolation facilities between 1 March 2020 and 30 April 2021 in Ibadan, Oyo state, Nigeria. From individual records, we evaluated patient demographics, COVID-19 risk factors, diagnostic testing, disease severity and antimicrobial utilization. The primary aim was to determine the prevalence of antimicrobial prescription as well as factors associated with antimicrobial prescribing in hospitalized patients with COVID-19 in Oyo state.

**Results:**

In total, 271 patients were included in this study. The median age of the population was 51 years (IQR; 32–62 years). The mean duration of hospital admission was 13 days (IQR: 10–14 days). Majority of participants were symptomatic (81.5%). All participants had a COVID-19 PCR test performed and none had bacterial culture performed. All patients received antimicrobial therapy across the entire cohort. The mean DOT per LOT across cohorts was 1.2 for mild cases, 1.4 for moderate cases and 1.3 for severe cases. Factors associated with the number of antimicrobials per prescription were being single (P = 0.02), being below 60 years of age (P = 0.04), mild COVID-19 symptoms (P < 0.001) and diabetes comorbidity (P = 0.03).

**Conclusion:**

Given the high rate of antimicrobial prescription and absence of bacteriological culture analysis in these patients, there is risk of development and spread of antimicrobial resistant. Continuous review of antimicrobial prescription is critical in the management of hospitalized COVID-19 patients.

## Background

Severe acute respiratory syndrome coronavirus 2 (SARS-CoV-2) also known as coronavirus disease 2019 (COVID-19) was first identified in Wuhan, China, at the end of 2019. Since then, the disease has spread affecting all countries across the world [1]. As of June 7 2020, more than 7 million cases of infection were identified worldwide and Africa was reported to be one the highest hit with 54,000 cases [1]. Nigeria reported the first index case of COVID-19 on 27 February 2020. By the next three months, more than 12, 000 cases were identified, putting Nigeria in the category of high risk African countries coupled with the weak state of the health system [2]. As of December 2020, approximately 84, 414 cases have been identified with 1,254 deaths [3]. According to the Nigeria Center for Disease Control and Prevention (NCDC) Interim Guideline for the Management of COVID-19, suspected case of COVID-19 is defined has an individual who presented with fever, cough, breathlessness, and who has a history of travel to any country with confirmed or ongoing transmission or close contact with a confirmed case or visited healthcare facility in which COVID-19 case is confirmed [4]. Probable case is defined as a suspected case, with ongoing or inconclusive laboratory findings. A confirmed case is defined as an individual with laboratory confirmed infection, with or without signs and symptoms [4]. The revised version of the interim guideline further defined probable case as any case where sample was not collected before the demise of the suspected case; suspected case as any healthcare worker with moderate to severe respiratory illness with prior contact with patient who present with respiratory symptoms with a prior history of travel abroad within 14 days.

The guideline states that all individuals with suspected and confirmed infection should be in isolation facilities with effective prevention measures. Suspected cases should be treated in separate rooms while confirmed mild and moderate cases should be admitted into the wards, and patients with severe COVID-19 should be transferred to the Intensive Care Unit (ICU) immediately [4]. It was further highlighted that prophylactic antimicrobial therapy should never be initiated in patients with asymptomatic or mild symptoms [4]. This guideline is similar to the WHO recommended guideline for clinical management of COVID-19 [5]. However, while the WHO highlighted 10days and 5days as the discharge criteria for symptomatic and asymptomatic patients respectively, the NCDC recommended 13days and 14days respectively. In addition, antimicrobial therapy are recommended for selected cases like severe disease, and moderate disease with comorbidity at risk of decompensation and should be the Access group (Access, Watch and Reserve ‐ AWaRe) [5].

Past influenza virus epidemics have revealed that early infection with *Staphylococcus pneumoniae* and *Haemophilus influenza* is associated with significant morbidity and mortality in infected patients [6, 7]. A review and meta-analysis study of COVID-19 patients in April, 2020 revealed bacterial co-infections in 7% and 8% of hospitalized patients and critically ill patients respectively [8]. Also, about 70% and over 90% of these patients were administered antimicrobial agents respectively [8, 9].

Despite the unprecedented pandemic, antimicrobial resistance (AMR) remains a hidden threat, accounting for approximately 3million cases and 35,000 deaths annually [10]. The lack of evidence-based guidelines on antimicrobial use in the beginning of the COVID-19 pandemic has led to widespread use in hospitalized COVID-19 patients [11]. While the use of antimicrobials in COVID-19 patients was considered not entirely irrational during the first wave, it is likely to have significant impact on AMR and antimicrobial stewardship (AMS) services. A survey of 86 respondents working in AMS revealed significant decrease in the mean impact of their AMS services during COVID-19 pandemic [12]. Although many studies have described antimicrobial consumption in hospitalized COVID-19 patients including inconsistencies between antimicrobial use and proven bacterial co-infection, understanding of antimicrobial consumption within geographical context is critical to inform national strategies targeted at promoting judicious antimicrobial use and reducing the spread of AMR. This study aimed to determine the prevalence of antimicrobial prescription as well identify factors associated with antimicrobial prescribing in hospitalized COVID-19 patients in Oyo state, Nigeria.

### Study design, setting and period

A retrospective study was performed on prescribed antimicrobial drugs to adult patients (aged ≥ 18years) with confirmed COVID-19 admitted across three isolation facilities between 1^st^ March 2020 and 30^th^ April 2021 in Ibadan, Oyo state, Nigeria. The state has a total of five isolation centers; Jericho Chest Hospital (Agbami), Ibadan, University College Hospital, Ibadan, Ladoke Akintola Teaching Hospital, Ogbomosho, Division 2 Nigeria Army, Odogbo, Ibadan, and Maternity Center, Olodo, Ibadan. Maternity Center, Olodo, also known as Infectious Disease Center is the largest isolation facility in the state. The other two largest facilities were Agbami Isolation Center and University Isolation Center, with a combined 15 bed space. Simple random sampling method was used to select three isolation centers including Olodo Isolation Facility, Agbami Isolation Facility and University College Hospital (UCH) Isolation Facility for this study.

### Population, sample size determination and sampling procedure

Sample size was determined using Yemane’s formula with margin of error (0.05) at 95% confidence interval (CI). The parameters include n = N/ (1+N (e2)). Where n = sample size, N = population size, e = acceptable error margin. The total number of hospitalized patients (aged ≥18 years) defined by *N*, admitted across isolation facilities in Oyo state was estimated to be 841. This gave a sample size of 271 hospitalized COVID-19 patients. We included patients who are ≥18 years of age with a confirmed case of COVID-19 (i.e. positive RT-PCR assay for SARS-CoV-2) diagnosed locally between 1^st^ March 2020 and 30^th^ April 2021. All patients with positive RT-PCR reports generated from hospital case notes in the study period were included in the study. Only local patients were included in the study in order to maintain uniformity of practice and to provide better understanding of local prescribing practices. Patients below 18years were not recruited into this study.

### Variables

Dependent variable was “antimicrobial utilization”. This include rate of antimicrobial utilization (number of participants with antimicrobial prescription), Days of Therapy, DOT (number of days that a patient received antimicrobial therapy regardless of the dose), Length of Therapy, LOT (number of days that a patient received antimicrobial agent irrespective of the number of different) and DOT per LOT (number of antimicrobial prescriptions per patient; ≤ 1 for monotherapy, > 1 for antimicrobial combination) [13]. The DOT per LOT was used to determine the factors associated with antimicrobial use.

The independent variables include; age (below 60 years, 60 years and above), sex (male, female), marital status (single, married, widow/divorced), comorbidity (no, yes), symptomatic (presence of fever, cough, fatigue), asymptomatic (absence of fever, cough, fatigue). COVID-19 severity (mild, moderate, severe) [4] and laboratory investigations (no, yes).

### Data collection

Between November 2021 and January 2022, data were collected from patients’ medical records using structured data spreadsheets. Data were first collected using a pretested structured spreadsheet. The spreadsheet was developed after reviewing different available literature. The data form captured patient demographics, COVID-19 risk factors, diagnostic testing (i.e., positive real time polymerase chain reaction [RT-PCR], respiratory culture, and respiratory virus pathogen panels), disease severity and antimicrobial utilization. Mild disease was defined as fever < 38°C, respiratory rate <30/min, peripheral oxygen saturation (SPO2) >93%, no shortness of breath, presence or absence of cough, no chronic disease comorbidity and no additional oxygen requirement; moderate disease was defined as respiratory rate >30/min, SPO2<93%, shortness of breath and need or increase need for supplemental oxygen; severe disease was defined as SPO2<88%, presence of comorbid conditions (such as diabetes, asthma, hypertension), severe respiratory distress, respiratory failure and need for invasive or non-invasive ventilation or intensive care unit (ICU).

Evaluation of antimicrobial prescribing was conducted to analyze prevalence of antimicrobial prescription, number of antimicrobial agents, spectrum of activity and duration of antimicrobial therapy. Antimicrobial utilization was evaluated by overall antimicrobial prescribing rate, prescribing rate per patient classification, prescribing rate per disease severity, prescribing rate by age, days of therapy (DOT) and DOT per length of therapy (LOT). To maintain quality of data and avoid biases, a properly designed data sheet was used and data collectors were trained. The data spreadsheet was pretested on 5% sample size and verified for completeness. The need for informed consent was waived by the Ethical Review Committee since the study data was collected retrospectively from patients’ medical records and patients’ identity were kept anonymous.

### Measurements

#### Days of therapy (DOT)

DOT is the number of days that a patient receives an antimicrobial agent regardless of the dose.

#### Length of therapy (LOT)

LOT is the number of days that a patient receives antimicrobial agent irrespective of the number of different antimicrobial agent used.

### Data processing and analysis

Data spreadsheets were reviewed and checked for completeness after data collection. Thereafter, data was coded and entered in SPSS version 17. Data were explored for missing values and treated accordingly. Descriptive statistics were used to present data using tables. Descriptive analysis included socio-demographic characteristics, symptoms and diagnosis, antimicrobial utilization.

In order to determine factors associated with antimicrobial utilization, bivariate analysis using chi-square was used to measure strength of association between outcome and independent variables. A p-value < 0.05 was considered as the cut-off point for declaring statistical significance.

### Ethical considerations

A written ethical approval letter was obtained from the University College Hospital Research and Ethical Review committee (Reference Number: NHREC/05/01/2008a).

## Results

### Demographic characteristics

A total of 271 participants were enrolled in this study. The median age of the population was 51 years (interquartile range (IQR); 32–62 years). Out of the 271 participants, majority were below 60 years (69%), male (62.3%) and married 56.0 (25.3). Majority were symptomatic (81.5%): 135.0 (61.1) cases were mild, 30.0 (13.6) were moderate and 31 (14.6%) cases were found to be severe. Patients below 60 years of age were more likely to experience severe symptoms compared with patients below 60 years. All participants had a COVID-19 PCR test performed. None had IgM, FBC, RVPS or Bacterial culture performed. (**Table 1)**.

**Table 1:** Demographic characteristics of study participants by symptom classification (N = 271).

Variables		Total n (%)	Asymptomatic n (%)	Symptomatic n (%)		
**Age group**		**271.0 (100.0)**	**50.0 (18.5)**	**Mild** **135.0 (61.1)**	**Moderate** **30.0 (13.6)**	**Severe** **56.0 (25.3)**
	**Below 60 years**	**187.0 (69.0)**	**31.0 (62)**	**104 (77.0)**	**9.0 (30.0)**	**43.0 (76.8)**
	**60 years and above**	**84.0 (31.0)**	**19.0 (38.0)**	**31 (23.0)**	**21.0 (70.0)**	**13.0 (23.2)**
**Sex**						
	**Male**	**170.0 (62.3)**	**48.0 (96.0)**	**82.0 (60.7)**	**8.0 (26.7)**	**32.0 (57.1)**
	**Female**	**101.0 (37.3)**	**2.0 (4.0)**	**53.0 (39.3)**	**22.0 (73.3)**	**24.0 (42.9)**
**Marital status**						
	**Single**	**62.0 (22.9)**	**12.0 (24.0)**	**34.0 (25.2)**	**7.0 (23.3)**	**9.0 (16.1)**
	**Married**	**201.0 (74.2)**	**38 (76.0)**	**100.0 (74.1)**	**20.0 (66.7)**	**43.0 (76.8)**
	**Widow/Divorced**	**8.0 (2.9)**	**0.0 (0.0)**	**1.0 (0.7)**	**3.0 (10.0)**	**4.0 (7.1)**

Freq. = Frequency, Perc. = Percentage, %, N = number of patients.

### Co-morbidity and days of hospitalization

Approximately 60.0% of participants had no comorbidity while 40.0% had one or more comorbidities at the point of COVID-19 infection diagnosis. Hypertension and diabetes were the most common pre-existing co-morbidities among patients. Participants with more than one co-morbidity tend to experience severe COVID-19 infection symptoms. The mean duration of hospital admission was 13 days (IQR: 10–14 days). (**Table 2**).

**Table 2:** Participants’ comorbidity and median days of hospitalization.

Variables		Total n (%)	Asymptomatic n (%)	Symptomatic n (%)		
		271.0 (100.0)	50.0 (18.5)	Mild 135.0 (61.1)	Moderate 30.0 (13.6)	Severe 56.0 (25.3)
**Have a Co-morbidity?**						
	**No**	163.0 (60.0)	34.0 (68.0)	88.0 (65.2)	8.0 (26.7)	33.0 (59.0)
	**Yes**	108.0 (40.0)	16.0 (32.0)	47.0 (34.8)	22.0 (73.3)	23.0 (41.0)
**Number of Co-morbidity**						
	**One**	86.0 (79.6)	14.0 (13.0)	39.0 (36.1)	16.0 (14.8)	17.0 (15.7)
	**Two**	14.0 (13.0)	1.0 (0.9)	7.0 (6.5)	3.0 (2.8)	3.0 (2.8)
	**Three**	8.0 (7.4)	1.0 (0.9)	1.0 (0.9)	3.0 (2.8)	3.0 (2.8)
	**Hypertension**	58.0 (53.7)	8.0 (7.4)	24.0 (22.2)	7.0 (6.5)	19.0 (17.6)
	**Diabetes**	42.0 (38.9)	4.0 (3.7)	21.0 (19.4)	13.0 (12.0)	4.0 (3.7)
	**Others**	8.0 (7.4)	4.0 (3.7)	2.0 (1.9)	2.0 (1.9)	0.0 (0.0)
**Median days of hospitalization. Median (IQR)**				13.0 (10–14)	14.0 (9–14)	14.0 (13–14)

Freq. = Frequency, Perc. = Percentage, %, n = number of patients, IQR = interquartile range.

### Antimicrobial utilization

All patients received antimicrobial therapy across the entire cohort. The median DOT and LOT was 3 days (IQR; 3–10 days) and 3 days (IQR; 3–7 days) respectively. The DOT per LOT across cohorts was 1.2 (SD; 0.4) for mild cases, 1.4 for moderate cases and 1.3 for severe cases. Patients with mild COVID-19 symptoms tended to receive fewer antimicrobial agents compared to patients with moderate and severe symptoms (**Table 3**).

**Table 3:** Antimicrobial utilization.

Days of Therapy (DOT)		Mild	Moderate	Severe
Median (IQR)	3.0 (3–10)	3.0 (3–8)	10.0 (6–11)	10.0 (6–13)
**Length of days (LOT)**				
Median (IQR)	3.0 (3–7)	3.0 (3–5)	7.0 (4–8)	8.0 (5–11)
**DOT/LOT**				
Mean (SD)	1.2 (0.4)	1. 2 (0.4)	1.4 (0.4)	1.3 (0.4)

### Factors associated with antimicrobial utilization

From bi-variate analysis using chi-square, being single, being below 60 years, and having mild COVID-19 symptom were significantly associated with antimicrobial combination across patient classifications. (**Table 4**). On multivariate firth logistic regression, being married [Adjusted Odds ratio: 2.57; 95% Confidence Interval: 1.05–6.29; p-value < 0.04]. Also, having moderate [Adjusted Odds ratio: 4.71; 95% Confidence Interval: 2.22–9.98; p-value < 0.001] and severe symptoms [Adjusted odd ratio: 4.53; 95% Confidence Interval; 1.87–10.98, p-value = 0.001] is significantly associated with being prescribed more antimicrobial agent (**Table 5**).

**Table 4:** Factors associated with antimicrobial use (chi-square statistics).

	Less antimicrobial Combination (i.e. DOT/LOT ≤1) N (%)	More antimicrobial Combination (i.e. DOT/LOT >1) N (%)	p-values
Marital status			
Single	53 (27.7)	9 (11.3)	**0.02**
Married	133 (69.6)	68 (85.0)	
Widow/Divorced	5 (2.6)	3 (3.75)	
**Age**			
Below 60 years	139 (72.8)	48 (60.0)	**0.04**
60 years and above	52 (27.2)	32 (40.0)	
**COVID-19 Symptomatic Status**			
Symptomatic	142 (74.3)	79 (98.8)	**< 0.001**
Asymptomatic	49 (25.7)	1 (1.2)	
**COVID-19 Severity**			
Mild	105 (73.9)	30 (38.0)	**< 0.001**
Moderate	16 (11.3)	14 (17.7)	
Severe	21 (14.8)	35 (44.3)	

**Table 5:** Factors associated with antimicrobial use (multivariate firth logistic regression).

	**Adjusted Odds Ratio (aOR)**	**95% Confidence Interval (CI)**	**p-values**
**Marital status**			
Single	1.00		
Married	2.57	1.05–6.29	**0.04**
Widow/widower	1.37	0.25–7.51	0.72
**Age group**			
Below 60 years	1.00		
60 years or older	1.21	0.34–4.16	0.77
**Symptomatic for COVID-19**			
No	1.00		
Yes	0.83	0.03–27.55	0.92
**Severity of symptoms**			
Mild	1.00		
Moderate	4.71	2.22–9.98	**< 0.001**
Severe	4.53	1.87–10.98	**0.001**

## Discussion

COVID-19 pandemic has changed the course of antimicrobial prescription, necessitating the crucial role of antimicrobial stewardship teams in detecting and preventing adverse consequences of inappropriate antimicrobial use. Around 15 different antimicrobials, belonging to cephalosporins, sulphonamide, floroquinolones, macrolides, nitroimidazoles and penicillins-like class were found to be used across patient classification in this study. These antimicrobials include ceftriaxone, meropenem, ceftriaxone-sulbactam, cefuroxime, ciprofloxacin, levofloxacin, erythromycin, amoxicillin, azithromycin, metronidazole, gentamicin, amoxicillin-clavulanic acid and sulphamethoxazole-trimethoprime. This finding correlates with a study on rapid review of COVID-19 national guidelines in ten African countries which revealed more than 15 antimicrobial prescriptions in asymptomatic, mild, moderate and severe hospitalized COVID-19 patients irrespective of complications [14]. Our finding also showed that most antimicrobial agents prescribed in this study are under the WHO ‘watch’ and ‘reserve’ antimicrobial list of AWaRe classification. This may further complicate the AMR situation and contribute to the development of multi-drug resistant organisms. The rate of antimicrobial usage across patient classification was found to be 100% as all patients received antimicrobial treatment. Secondary analysis of eighteen studies reporting antimicrobial prescribing in patients with COVID-19 showed that more than 70% of patients received antimicrobial therapy with absence of prescribed antimicrobial stewardship intervention [15]. Similar rate of antimicrobial therapy was observed in other studies [9, 16]. The high rate of antimicrobial utilization may be due to suspected bacterial infection among physicians. This is in line with the prescription of World Health Organization (WHO) which advised against the use of antimicrobial therapy in COVID-19 patients unless there is suspicion of bacteria co-infection [17]. Also, the Nigerian Interim Guideline for management of COVID-19 patients which was later adopted also recommended empiric antimicrobial therapy in confirmed COVID-19 patients [18].

Our study revealed antimicrobial utilization in all patients without strong diagnostic evidence to distinguish between bacterial and viral infection. Asymptomatic as well as mild and moderate cases were found to receive antimicrobial therapy without diagnostic evidence of a bacterial co-infection. This finding resonates with a review on antimicrobial use in COVID-19 patients which revealed more than 50% antimicrobial prescription rate in mild and moderate cases [7]. Studies have shown that bacterial infection rate in COVID-19 Patients is less than 20% [7, 8, 19]. This situation establishes the likelihood of utilization of antimicrobial drugs even in hospitalized COVID-19 patients who otherwise do not need antimicrobial therapy. Although evidences have supported empirical use of antimicrobial drugs in COVID-19 patients due to longer turnaround time of bacterial culture results, increased utilization may increase risks of *Clostridioides difficile* infection and subsequent emergence and spread of multidrug resistance organisms [6, 11, 12, 20, 21], which can in turn have negative impact on prognosis and outcome of severe COVID-19 patients receiving emergency hospital care [22, 23]. In addition, the WHO highlighted that extensive antimicrobial use can lead to increased rate of bacterial resistance, which can increase the burden of morbidity and mortality COVID-19 patients during and after pandemic [5]. Efforts to obtain bacteriological culture are critical to ensure antimicrobial stewardship.

Our study revealed that patients with mild COVID-19 tend to receive fewer antimicrobial agents with a mean DOT per LOT of 1.2 compared to 1.4 and 1.3 for moderate and severe patients respectively. This finding is consistent with a study conducted at a Tertiary Care Center in Minnesota which showed that mild COVID-19 patients received fewer antimicrobial drugs compared to moderate and severe patients [24]. There is a need for strong diagnostic criteria to carry out all-inclusive microbiological and resistance analysis where there are existing laboratory diagnostic infrastructures in order to inform antimicrobial usage.

This study showed that age, marital status, COVID-19 symptom status and COVID-19 severity were significantly associated with antimicrobial utilization. Patient age was found to be significantly associated with fewer antimicrobial combination. Also, symptom classification, marital status and COVID-19 severity was found to be significantly associated with more antimicrobial combinations.

### Limitations

This study only covered hospitalized COVID-19 patients in Oyo state and did not provide expanded knowledge and understanding of antimicrobial utilization in wider populations. This study did not capture reasons for lack of bacteriological culture during admission. Further study is required to explore quality of care in terms of saturated hospital structure. Also, there a need for further investigations to understand the patients’ culture, distribution of antimicrobial agents across patient disease severity as well as why Nigeria diverged from WHO guidelines and the consequences. However, this study provided understanding of local antimicrobial utilization and revealed the level of antimicrobial prescribing practices in COVID-19 patients admitted across isolation facilities in Oyo state. The findings in this study can contribute to improved professional practices across Nigeria. In addition, findings in this study can be used to predict and influence future public health preparedness against antimicrobial resistance and antimicrobial stewardship policy.

## Conclusion

This study revealed several critical findings related to use of antimicrobial drugs in hospitalized COVID-19 patients. Given the high rate of antimicrobial prescription and lack of bacteriological culture analysis in these patients, there is risk of development and spread of antimicrobial resistance. Factors associated with antimicrobial use include age, symptom classification, marital status and disease severity. Future opportunities include improved diagnostics, evaluation of COVID-19 co-infections and identifying relevant antimicrobial stewardship initiatives in patients with COVID-19.

## Supporting information

S1 Checklist Inclusivity in global research. (DOCX)

S1 Data (XLSX)
